# Thermo-responsive circularly polarized luminescence from carbon quantum dots in a cellulose-based chiral nematic template

**DOI:** 10.1515/nanoph-2024-0091

**Published:** 2024-06-03

**Authors:** Haidong Shi, Jiaxin Zhu, Yaxuan Deng, Yanling Yang, Changxing Wang, Yihan Liu, Wanlong Zhang, Dan Luo, Da Chen, Yue Shi

**Affiliations:** School of Physical Science and Technology, 47862 Ningbo University, Ningbo, Zhejiang 315211, China; Department of Electrical and Electronic Engineering, 255310 Southern University of Science and Technology, Shenzhen, Guangdong 518055, China; Nanophotonics Research Centre, 47890 Institute of Microscale Optoelectronics, Shenzhen University, Shenzhen, Guangdong 518060, China

**Keywords:** circularly polarized luminescence, cholesteric liquid crystal, thermo-responsive, hydroxypropyl cellulose, carbon quantum dot

## Abstract

Circularly polarized light emitting active materials are of great interest, and the convenient tuning of the circularly polarized luminescence (CPL) remains a significant challenge. Integrating fluorescent materials into chiral photonic crystals to achieve tunable CPL is a promising approach, allowing efficient manipulation of CPL by adjusting the photonic band gap (PBG). We combined carbon quantum dots (CQDs) with hydroxypropyl cellulose (HPC), which self-assembles into a cholesteric liquid crystal (CLC). The helical structure can selectively reflect right circularly polarized (RCP) light, achieving strong circular dichroism (CD) and high CPL dissymmetry factor *g*_lum_. In addition, the chiral template is thermo-responsive. The CPL wavelength can be adjusted by regulating the PBG position through temperature adjustment, while the chirality of CPL keeps high especially in the heating process. This work enables stimuli-responsive manipulation of CPL under one template through temperature regulation, which may open up enormous possibilities for the cellulose-based material in different areas.

## 1 Introduction

Circularly polarized light has unique optical properties and broad application potential in optical storage devices, 3D displays, and other fields [1], [2], [3], therefore, attracting great attention in recent years. CPL systems have been developed in various systems, including chiral organic light emitters [4], [5], conjugated polymers [6], chiral supramolecules [7], [8], [9], chiral nanomaterials [10], [11], [12], [13], and chiral template [14], [15], [16]. However, most systems have disadvantages such as cumbersome synthesis and preparation process, weak CPL dissymmetry factor, etc. [17]. Among them, co-assembling fluorescent materials with chiral template hosts is simple and effective [14], [15], [16]. The produced CPL depends on the property of the chiral template hosts, allowing the incorporation of various achiral luminophores to give chiral signal. Among various chiral templates, CLC, a typical liquid crystal (LC) state with a periodic 1D helical structure, provides one of the most effective chiral templates on the realization of CPL [18], [19], [20], [21], [22], [23]. The helical structure works as a 1D chiral photonic crystal, allowing efficient manipulation of spontaneous emission within its PBG to give high CPL signal and tunability. However, most tunable CPLs were achieved using thermotropic CLCs [19], [20], [21], which are usually make up of low-molecular-mass materials that faces the compatibility issues due to phase separation, especially with the novel fluorescent nanomaterials such as upconversional nanoparticles [24], [25], quantum dots [26], [27], perovskites [28], and so on.

As a result, attention has been turned to lyotropic LCs of macromolecules that could form CLC states as chiral templates. Such templates not only allow for high CPL dissymmetry factor *g*_lum_ of CPL but also offer good compatibility with various fluorescent nanomaterials. For example, cellulose nanocrystals (CNCs) that could self-assemble into a 1D helical structure as a chiral template has been doped with different fluorescent materials to achieve CPL in recent years [29], [30], [31], [32], [33]. However, the CNCs must be dried to form PBG in the visible range. Although the PBG of CNC template could be adjusted by changing concentration [34], ultrasonication [35], and adding salts before drying up [36], once the solid film is formed, it is basically fixed. The tunability was reported by adding responsive additives to render the film stimuli-response to humidity and acid/base [37], [38]. However, the adjustment range was limited and the dissymmetry factor *g*_lum_ was lowered by the additives. To achieve tunable CPL in real time with high *g*_lum_ is important for developing advanced intelligent CPL systems based on lyotropic LC of macromolecules.

HPC is an important cellulose derivative with lyotropic LC property [39], [40], [41]. Its aqueous solution can self-assemble into a right-handed CLC phase, selectively reflecting RCP light [42]. More importantly, HPC aqueous suspension is temperature-responsive. An increase in temperature causes a red-shift in its PBG position [43], [44]. Therefore, when incorporated with fluorescent materials, HPC may offer a great chiral template to achieve tunable CPL with response to temperature. Herein, we introduce CQDs into the HPC chiral template to impart chirality to its luminescence. CQDs are novel fluorescent materials with tunable photoluminescence, high emission intensity, good photostability, and good biocompatibility, showing broad prospects in sensing, light-emitting diodes, energy storage, and optoelectronic applications [45], [46], [47], [48]. The HPC template renders the chirality to luminescence based on lyotropic LCs of macromolecules, offering a wide-ranged thermo-responsive CPL property in real time with much higher dissymmetry factor *g*_lum_ compared to other systems [49], [50], [51], [52].

## 2 Experimental section

### 2.1 Materials

HPC powder (viscosity of 4.4 mPa⋅s in 20 °C/2 %, aqueous solution) was purchased from Nisso, Japan. Levofloxacin was purchased from Aladdin.

### 2.2 Preparation of CQDs

The CQDs were synthesized using a simple one-step hydrothermal method [45]. First, 1 mmol levofloxacin was dissolved in 10 mL deionized water by ultrasonic treatment. Then, the mixture was transferred to a 100 mL Teflon-lined stainless-steel autoclave and heated at 200 °C for 8 h. After cooling to room temperature naturally, the obtained solution was filtered through a 0.22 μm membrane to remove unreacted raw materials and impurities and then purified by dialysis using a dialysis membrane with a molecular weight cut-off at 1,000 Da for 1 week. Finally, the solution was freeze-dried into powder and stored at room temperature.

### 2.3 Preparation of HPC/CQD composite

Certain amounts of CQDs (0.4 μg, 0.8 μg and 1.2 μg) in 1 mL of deionized water were mixed thoroughly with 2.3 g of HPC powder. The mixture was then centrifuged at a speed of 10,000 rad/min for 20 min to eliminate the air bubbles generated during the mixing process. After that, the HPC/CQD composite material was transferred to a quartz cuvette with 1 mm optical path and left for at least 1 h for self-assembly. The above preparation processes were performed at room temperature of about 20 °C, followed by further thermo-responsive characterizations at different temperatures.

### 2.4 Characterization

The fluorescent images of the HPC/CQD material were taken under a 365 nm UV lamp irradiation (Baoshan, ZF-7B). Photoluminescence (PL) and photoluminescence excitation (PLE) spectra were collected at room temperature by a luminescence spectrometer (PerkinElmer, LS55). The ultraviolet–visible (UV–vis) absorption spectrum was characterized by a UV-5800 spectrophotometer. For Fourier transform infrared spectra (FTIR)  characterization, the HPC powder was mixed with KBr, grounded, and pressed into tablet for FTIR testing (Thermo Scientific, Nicolet 6,700), while CQDs and HPC/CQD samples were freeze-dried and then ground into powder before adding into KBr. The CD spectra were measured using a circular dichroism spectrometer (Jasco, J-1700), and the CPL properties were measured using a CPL spectrometer (Jasco, CPL-300) at different temperatures. The reflection spectra of the samples were recorded by an optical microscope (Nikon, E600 POL) equipped with a thermally controlled stage (Instec, mK2000B) and a UV–Vis spectrometer (Ocean Optics, USB2000+). For scanning electron microscopy (SEM) and energy dispersive X-ray spectroscopy (EDX) characterizations, the HPC/CQD material was dried into a thin film and then immersed in liquid nitrogen and brittle-fractured for cross-sectional characterization on a Hitachi SU-70 system after Pt coating. The morphology of HPC/CQD composite material was characterized using transmission electron microscopy (TEM, Hitachi HT-7800). Time-resolved photoluminescence (TRPL) was measured with a time-resolved fluorescence spectrometer (Edinburgh, FLS980).

## 3 Results and discussion

### 3.1 Properties of HPC/CQD composite materials

Fluorine-containing quantum dots have high fluorescence quantum yield and stability because fluorine atoms can inhibit defects and oxidation on the surface of the quantum dots, thus improving their luminescence efficiency and lifetime. Previous work of our coauthors revealed that such CQDs are spherical and monodispersed through TEM characterization [45]. The average diameter is 3.9 nm, and the high-resolution TEM (HR-TEM) image clearly showed that the lattice spacing of the (100) facet is about 0.21 nm, indicating a graphite-like structure. FTIR and X-ray photoelectron spectroscopy revealed that there are a large amount of C–N, –OH, and C–F groups on the surface of CQDs, allowing their hydrophilic property [45].

**Figure 1:**
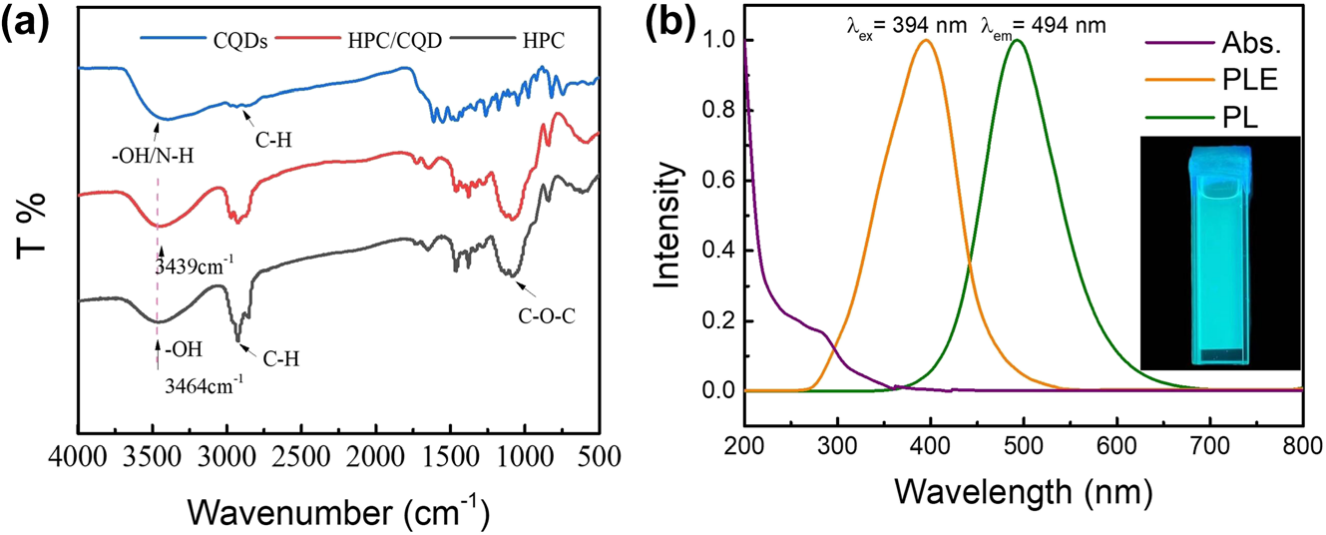
Optical characterizations. (a) FTIR spectra of CQDs, HPC, and HPC/CQDs. (b) Normalized UV–vis absorption, maximum excitation, and maximum emission spectra of HPC/CQD material (0.4 μg CQDs). Inset: Fluorescence image of HPC/CQDs in a quartz cuvette under UV irradiation.

To clarify the interaction between HPC and CQDs, the FTIR of CQDs, HPC, and HPC/CQDs were measured and shown in Figure 1(a). After adding CQDs to HPC, the typical FTIR peaks of cellulose exist (Figure 1(a)), such as the stretching vibration absorption peak of C–H of the primary carbon near 2,924 cm^−1^ and the C–O–C stretching vibration peaks between 1,200 and 1,000 cm^−1^ [53], indicating the introduction of CQDs does not affect the characteristic functional groups of HPC. On the other hand, the large number of C–N and C–F surface groups of CQDs facilitate the formation of hydrogen bonds [45], which averages out the electron cloud density and results in a decrease in the stretching vibrational frequency of hydroxyl groups from 3,464 cm^−1^ to 3,439 cm^−1^, ensuring the stable and homogeneous mixing of the HPC/CQD composite material. This is also confirmed by the TEM images of the HPC/CQD samples in Figure S1, where the CQDs are well dispersed in the HPC matrix.

The CQDs have a concentration-dependent PL property [45]. When the CQDs were doped with HPC at different concentrations (0.4 μg, 0.8 μg, and 1.2 μg), different PL behavior was also presented, where the 2D excitation–emission maps are shown in Figure S2. All the composite materials exhibit an excitation-dependent PL feature. Take the 0.4 μg CQD sample for example (Figure S3). The maximum excitation wavelength (*λ*_ex_) of the HPC/CQD composite material is around 395 nm, and the maximum emission wavelength (*λ*_em_) is at 494 nm (Figure 1(b)) (luminescence lifetime is given by TRPL in Figure S4). The composite material shows strong absorption in the UV region, giving little disturbance on subsequent optical tests. Under UV illumination, the HPC/CQD composite material exhibits cyan fluorescence color (the inset of Figure 1(b)). It can be seen that the sample is homogeneous without aggregation, further confirming that the CQDs are well dispersed in the HPC matrix.

**Figure 2:**
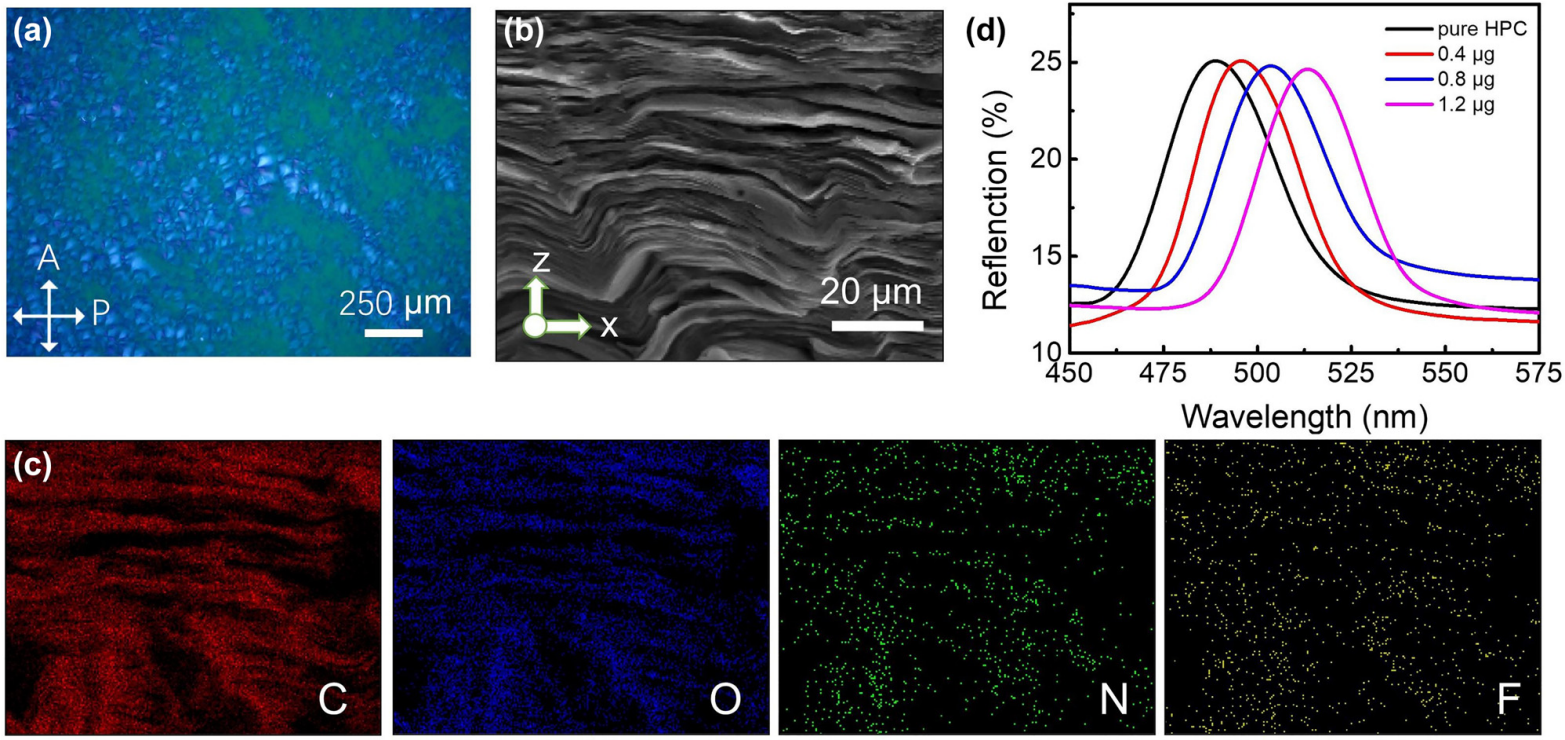
Structure of HPC/CQD composite materials. (a) Transmissive POM image and (b) cross-sectional SEM image of an HPC/CQD composite film with (c) EDX maps. (d) Reflection spectra of HPC/CQD composite materials with different amounts of CQDs.

After 72-h self-assembly of the HPC/CQD composite material at room temperature, planar and focal conic textures can be observed under polarized optical microscope (POM), as shown in Figure 2(a), indicating that HPC/CQD composite material self-assembles into a CLC phase. To observe the internal structure, the dried composite film was fractured to expose the cross section for SEM observation. The quasi-layer structure was observed as shown in Figure 2(b), demonstrating the CLC structure of the HPC/CQD composite material. The EDX maps of the HPC/CQD samples show that the specific F and N elements of CQDs are uniformly distributed in the CLC structure as shown in Figure 2(c), indicating that CQDs are well dispersed in the HPC matrix. Since the periodic helical structure of CLC phase resembles the chiral photonic crystal, the HPC/CQD composite materials reflect the light as its chirality within the PBG. The reflection spectra of three HPC/CQD composite materials with different amounts of CQDs are shown in Figure 2(d). The peak position *λ*_0_ gradually red-shifts from 488 nm to 513 nm with increasing CQDs amount, indicating an increase in the helical pitch due to the insertion of CQDs. Here, the helical pitch *P* could be estimated from the De Vries formula *λ*_0_ = *n·P·*cos*θ* [54], where *n* is the average refractive index of HPC suspension of about 1.485–1.533 [55], [56], [57], and *θ* is the angle between the reflected light and the cholesteric helical axis.

### 3.2 Thermo-responsive HPC/CQD composite materials

**Figure 3:**
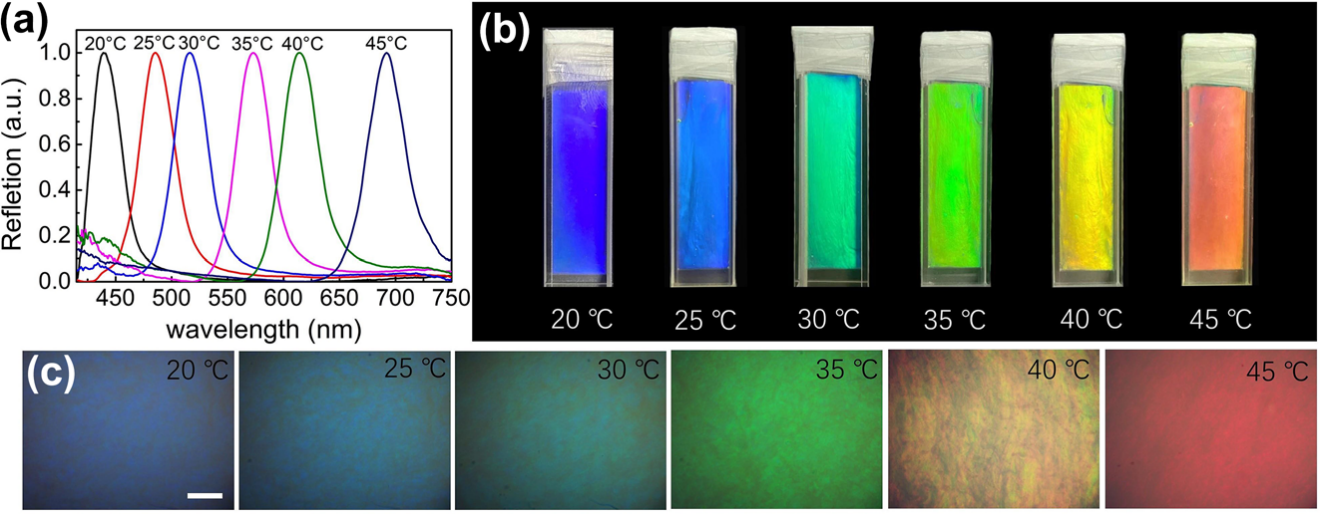
Thermo-responsive selective reflection of  HPC/CQD composite materials. (a) Normalized reflection spectra of HPC/CQD composite materials at different temperatures. (b) HPC/CQD material in a cuvette observed at different temperatures under nature light. (c) Microscopic images of corresponding sample observed under reflective microscope. Scale bar is 250 µm.

In addition to the lyotropic LC phase, HPC has attracted much attention due to its unique thermo-responsive property. When it self-assembles into CLC phase, an increase in temperature causes a red-shift in its PBG position [43], [44]. The increase of the helical spacing during heating is due to the hydrogen bond breaking between HPC and water, which increases the intermolecular repulsive force under the effect of high temperature [44]. Therefore, when incorporated with CQDs, HPC may offer a great chiral template to achieve tunability of CPL with response to temperature. To test this, the HPC/CQD sample (take 0.4 μg CQDs for example) was heated from 20 °C to 45 °C. The reflection wavelength of the HPC/CQD material red-shifts as temperature increases (Figure 3(a)). The reflected color of the chiral HPC/CQD material comes from the PBG of a periodic helical structure of CLC, showing a characteristic iridescence as shown in Figure 3(b). When the temperature is 20 °C, the reflection wavelength is about 434 nm. The reflection color of HPC/CQD is blue-violet, as shown in the macroscopic picture in Figure 3(b) and the reflective microscopic image in Figure 3(c). When the sample is raised to 25 °C, the reflection wavelength red-shifts to 485 nm, and the reflection color turns blue. When the temperature further rises to 30 °C, 35 °C, and 40 °C, the corresponding reflection wavelength increases to 516 nm, 573 nm, and 614 nm, and the reflection colors becomes cyan, green, and yellow. When the sample temperature rises to 45 °C, the reflection wavelength increases to 692 nm and the reflection color is red. The reflection wavelength shifts by 258 nm during the temperature increasing process from 20 °C to 45 °C, covering almost the entire PL spectrum range of CQDs (Figure 1(a)). The reflective POM images of the HPC/CQD samples at different temperatures are shown in Figure S5. If the temperature keeps rising, the reflection spectrum will continue to red-shift outside the PL spectrum and, therefore, does not show here. When the temperature increases to 55 °C, the lower critical solution temperature (LCST) of HPC suspension is reached above which a phase separation happens and isotropic domains start to appear, resulting in a reduction in transmittance and should be avoided in this application [39], [40].

### 3.3 Tunable optical activity under temperature stimulation

**Figure 4:**
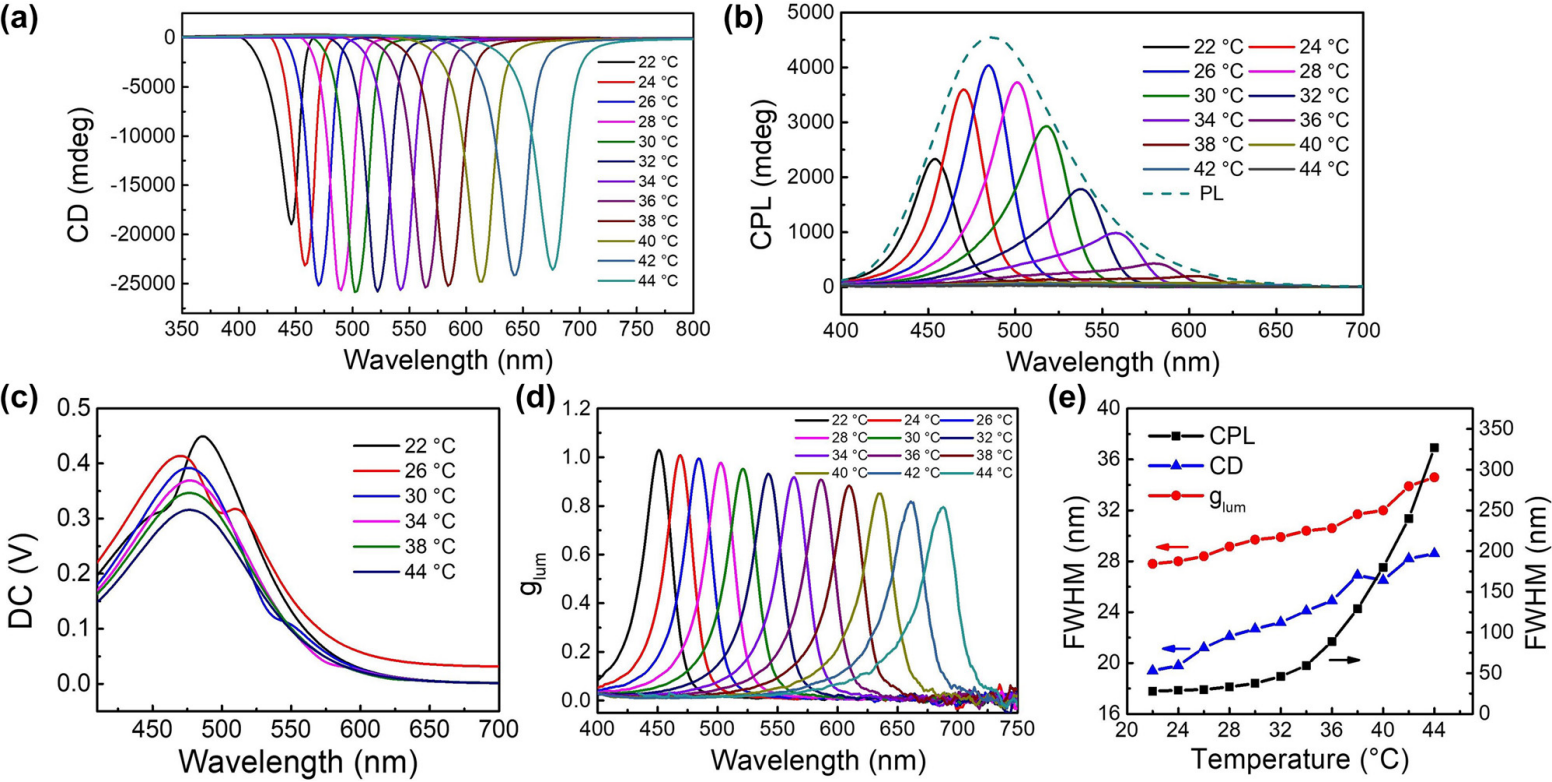
Thermo-responsive optical activities of the HPC/CQD composite material. (a) CD spectra, (b) CPL spectra, (c) DC spectra, and (d) *g*_lum_ spectra of the HPC/CQD composite material at different temperatures under 360 nm UV excitation. (e) Full width at half maximum (FWHM) of CD, CPL, and *g*_lum_ spectra at different temperatures.

The PBG of the CLC photonic crystal prohibits the passage of the light with the same helicity inside, causing the extinction difference between left circularly polarized (LCP) light and RCP light, which is presented by the CD spectrum. Since HPC has a right-handed CLC structure, the HPC/CQD material selectively reflects the RCP light and gives a negative CD signal. The CD spectra at different temperatures were investigated as shown in Figure 4(a). At starting temperature of 22 °C, the peak value of CD intensity was around −19,000 mdeg. The absolute value of CD intensity gradually increases when the temperature rises until about 30 °C, where the CD intensity was the strongest, reaching over −25,000 mdeg. Then the absolute value decreases slowly but still keeps high. For comparison, the CD spectra of different CQD contents are shown in Figure S6. All the samples have similar CD spectra as described above with the highest CD intensity reaching over −25,000 mdeg, indicating that the HPC/CQD composite material maintains a regular CLC structure during heating process. More importantly, the CD spectra gradually red-shift with increasing temperature, the same as the reflection spectra at elevated temperatures (Figure 3(a)), indicating a thermo-responsive PBG shift.

Since HPC has a right-handed chiral structure, the RCP light is prohibited inside the material while the LCP light can propagate, causing the chiral dissymmetry of the luminescence in the excited state. As shown in Figure 4(b), HPC/CQD composite material produces a stronger LCP luminescence under UV irradiation, giving a positive CPL. When the temperature increases from 20 °C to 26 °C, the CPL wavelength red-shifts and the intensity is significantly enhanced. This is due to the bathochromic PBG position during the temperature increase process, resulting in the better overlapping of PBG and PL spectrum and thus the stronger CPL intensity. When the temperature continues to increase, the PBG continues to red-shift and gradually moves away from the PL peak. This causes the red-shift of the CPL wavelength and the gradual decrease of the intensity. When the temperature increases to 44 °C, the PBG position almost reaches the edge of the CQDs’ PL spectrum and the CPL intensity becomes very weak. The envelope of the thermo-responsive CPL spectra keeps consistent with its PL spectrum as shown in Figure 4(b). Therefore, the modulation of CPL position can be achieved by adjusting the PBG position at different temperatures, and the CPL intensity is related to the overlap degree between the PBG and the PL spectra.

Although the composite material exhibits thermo-responsive CPL emission, the DC spectra, which are the modulated PL spectra, do not vary much as shown in Figure 4(c). Different dips are due to the selective reflection of the helical structure, and the overall intensity drop is probably due to the light scattering with increasing temperature. The PL spectra of CQDs stay the same at different temperatures, as shown in Figure S7, indicating that the fluorescence property of CQDs does not change with temperature. Noted that the PL spectrum is much broader than the PBG of the HPC/CQD composite material, whose FWHM is about 20 nm, allowing the PBG to be adjusted freely within the PL range to produce tunable CPL.

To explore the chirality of CPL, the CPL dissymmetry factor defined as *g*_lum_ = Δ*I*/*I* = 2(*I*_L_ − *I*_R_)/(*I*_L_ + *I*_R_) was evaluated (Figure 4(d)), where *I*_L_ and *I*_R_ denote the LCP and RCP luminescence intensity, respectively. The *g*_lum_ is a crucial parameter for measuring the optical activity of CPL. At the starting temperature of 22 °C, the PBG peak of the HPC/CQD material is at the blue edge of the PL spectrum, and the *g*_lum_ intensity is 1.02. As temperature increases, the *g*_lum_ red-shifts while the value decreases. Although the overlapping area of the PBG with the PL spectrum gradually increases and then decreases across 26 °C, the *g*_lum_ continues to decrease with increasing temperature. It is contrary to what one might expect that the strongest *g*_lum_ should appear when the PBG coincides with the PL spectrum [36]. Noted that the FWHMs of CD, CPL, and *g*_lum_ spectra all broaden as temperature increases as shown in Figure 4(e), indicating a less ordered CLC structure and a stronger thermal motion of HPC molecules at higher temperature. This may be the reason why the *g*_lum_ value decreases with increasing temperature [21]. However, even when the temperature rises to 44 °C where the PBG is at the red edge of the PL, the *g*_lum_ is still as high as 0.8, indicating that the cholesteric structure of HPC provides a good thermal-responsive template for regulating the light chirality during the heating process.

**Scheme 1:**
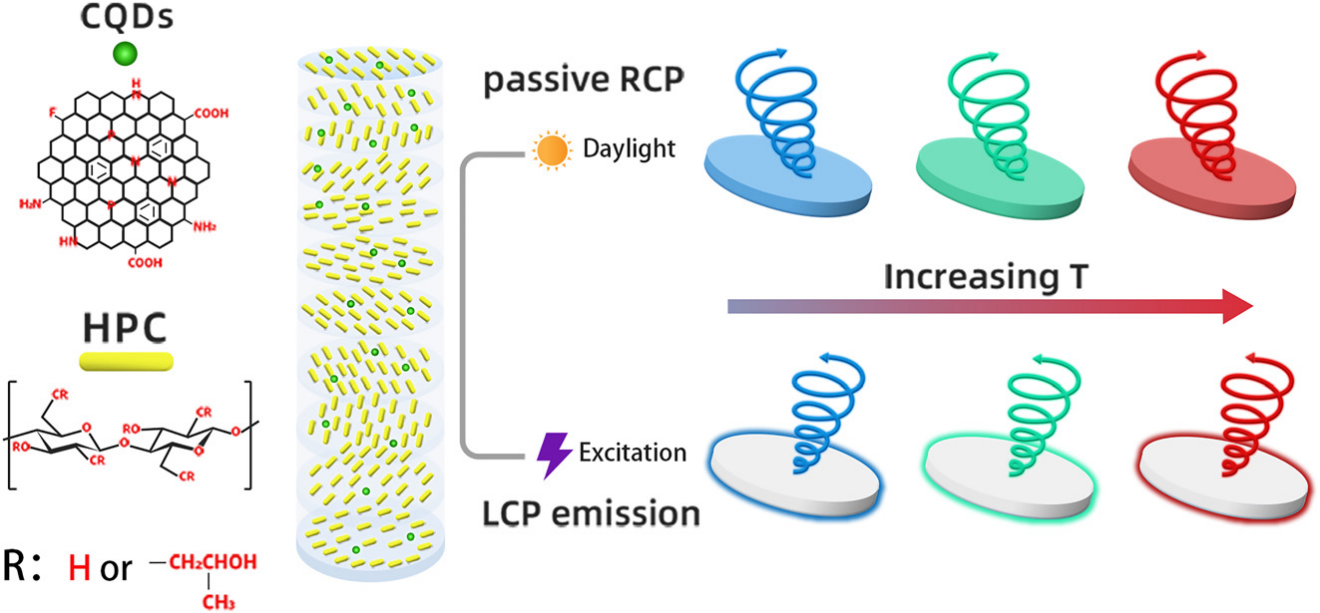
Schematic diagram of the thermo-responsive CPL from the HPC/CQD material.

The schematic diagram of the thermo-responsive CPL is shown in Scheme 1. The HPC/CQD composite material selectively reflects RCP light under daylight and emits LCP fluorescence under excitation, where the PBG position show a thermochromic shift with increasing temperature to give a thermo-responsive optical activity.

Previous study showed that the CQD solution had an excitation-dependent PL property [45]. The HPC/CQD material also has an excitation-dependent PL property as shown in Figure S3. Besides 360 nm, 395 nm, and 410 nm were selected as excitation wavelengths. The CPL spectra shift for different excitation wavelengths but consistent with the excitation-dependent PL spectra (Figure S8). More importantly, the composite material has similar *g*_lum_ spectra with different excitation wavelengths (Figure 4 and Figure S8). Therefore, to avoid the spectrum cut near the excitation region, the UV excitation is suggested for the optical activity exploration. On the other hand, the CPL spectra of different CQD contents are shown in Figure S6. The envelope of each thermo-responsive CPL spectra keeps consistent with its corresponding PL spectrum. And all the samples have similar *g*_lum_ spectra as described above, indicating that the HPC/CQD composite material maintains a regular CLC structure at our doping extent.

**Figure 5:**
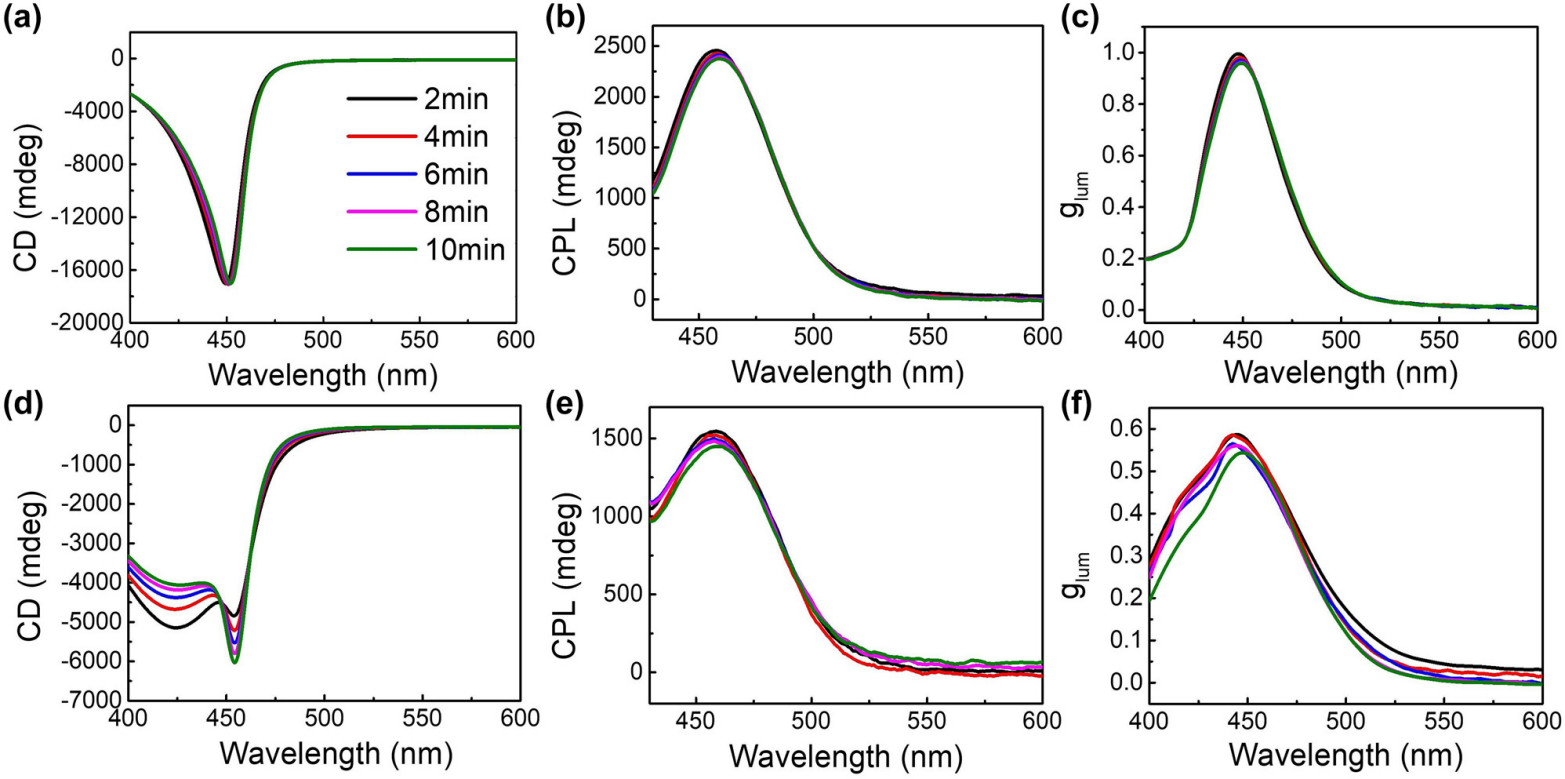
Optical activities of the HPC/CQD composite material at different relaxation times. (a) CD, (b) CPL, and (c) *g*_lum_ spectra of HPC/CQD composites maintained at 22 °C for different times by heating directly from 15 °C to 22 °C. (d) CD, (e) CPL, and (f) *g*_lum_ spectra of the HPC/CQD composite after direct cooling from 30 °C to 22 °C and maintained for different times.

Noted that the viscosity of HPC suspension is high, especially for the high-concentrated HPC in CLC phase [58]. During the heating process directly from 15 °C to 22 °C, the optical activity was explored as a function of time as shown in Figure 5(a)–(c). All the CD, CPL, and *g*_lum_ spectra show quick response during heating process, giving high values within 2 min. Quicker response probably applies but limited by the relatively slow CPL measuring process. However, the case is different for the cooling process. As shown in Figure 5(d), after direct cooling from 30 °C to 22 °C, except for the main peak at the original place, the CD spectra show a broad shoulder at shorter wavelength. Although the shoulder becomes smaller with time, it still exists after longtime relaxation. The CPL and *g*_lum_ spectra in Figure 5(e) and (f) show the same behavior, where the FWHMs expand after cooling, especially at shorter wavelength region. This may be explained by undulation instability in viscous material with periodic structure. The helical pitch shrinks during direct cooling. In the high viscous medium, the strain could be relaxed by tilting the helical axis with constraint by the cell surface, giving a periodic undulation [42], [59], [60]. In this way, defects could be avoided by introducing splay deformation of the helical axis to reduce the free energy [61], [62], [63]. This phenomenon could be noticed in the cross-sectional SEM image of an HPC/CQD composite film as shown in Figure 2(b), where shrinkage of the helical pitch could not be avoided during the preparation process, therefore, causing the periodic structure tilting and undulating, which were commonly observed in the SEM images showing CLC phase in previous reports [42], [44]. According to the De Vries formula, the reflected wavelength decreases with tilted helical axis due to the increasing angle *θ*, which may explain the broader FWHM and the shoulders observed at shorter wavelength as shown in Figure 5(d)–(f). Quantitative analysis needs to be further explored in future work. However, although the CPL gets worse during the cooling process, the dissymmetry factor *g*_lum_ is about 0.55, which is still much larger than those of most stimuli-responsive CPL reported at present, especially for the systems which has good compatibility with nanoparticles (Table S1).

### 3.4 Relationship between CPL and HPC concentration

**Figure 6:**
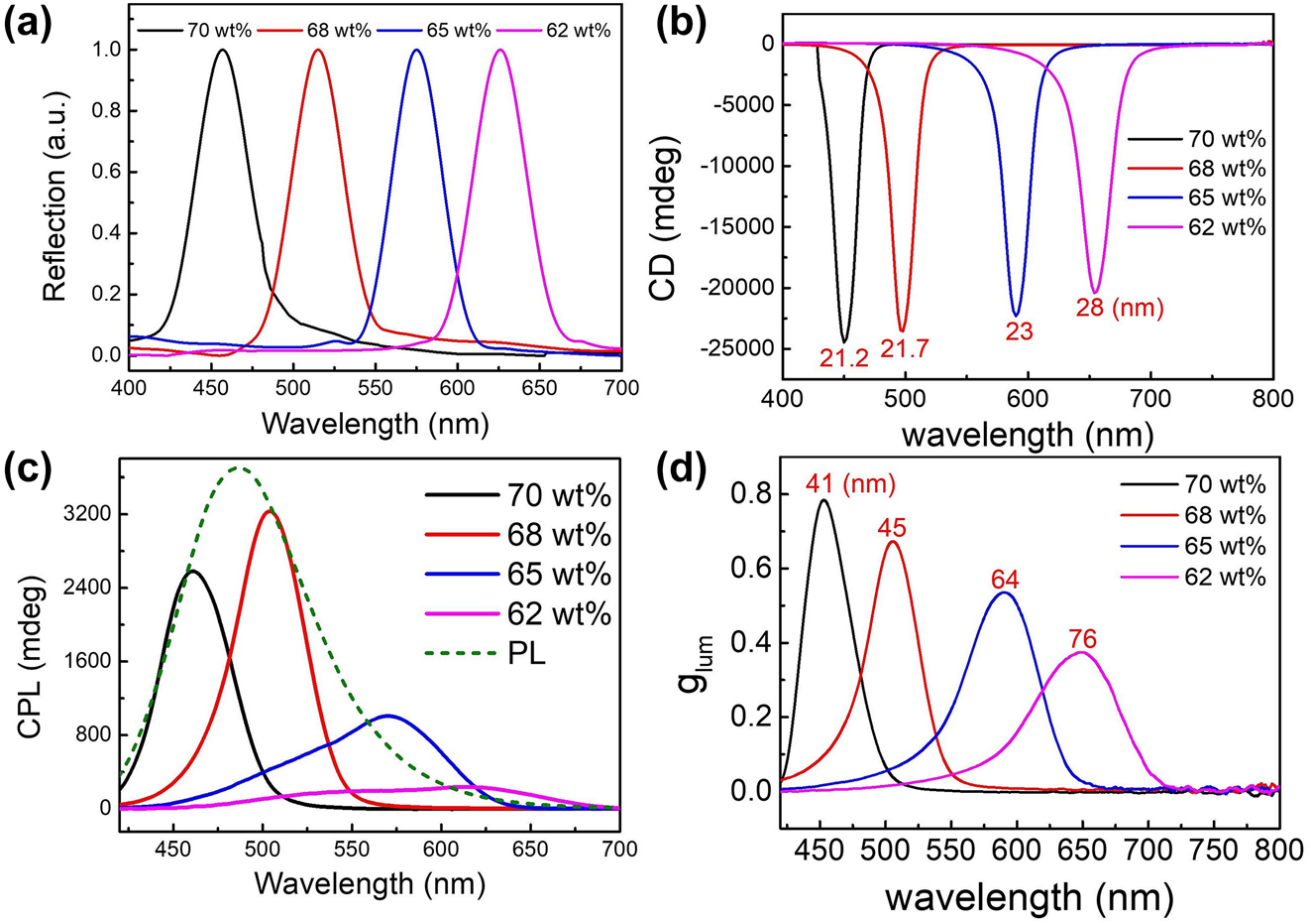
Optical characterizations of the HPC/CQD composite materials with different HPC concentrations. (a) Reflection spectra, (b) CD spectra, (c) CPL spectra, and (d) *g*_lum_ spectra of the HPC/CQD composite materials with different HPC concentrations. The FWHMs are indicated in red numbers in (b) and (d).

To clarify the reason for the decrease of *g*_lum_ during the heating process, the relationship between the chiral structure of HPC/CQD material the *g*_lum_ value was investigated with different HPC concentrations. The HPC-to-CQD ratio was kept the same as before, and the samples were investigated at 25 °C to exclude the temperature effect. Different HPC concentrations give different helical pitches, and thus the PBG position can be tuned by HPC concentration. The PBG red-shifts with lower HPC concentration, indicated by the reflection and CD spectra in Figure 6(a) and (b). As a result, the CPL spectrum red-shifts. The CPL intensity increases as the PBG approaches the PL peak and decreases as the PBG moves away (Figure 6(c)). The strongest CPL intensity was found in the 68 wt% HPC/CQD sample with the closest PBG position to the PL peak, which is the same as the temperature-dependent trend discussed above. However, the chirality of CPL, indicated by the *g*_lum_ value, only depends on the CLC order. The FWHMs increase with the red-shift of PBG, which are marked in Figure 6(b)–(d) in both CD and *g*_lum_ spectra, indicating that the CLC order decreases with longer helical pitch [21]. A longer helical pitch results in a lower CLC order, that is, a less regular chiral structure, giving a lower CPL chirality, indicated by a decreasing *g*_lum_ value as shown in Figure 6(d). This is consistent with the trend caused by temperature. Therefore, when the narrowband chiral template of HPC is combined with a broadband fluorescent material, the chirality of CPL could be controlled by either HPC concentration or temperature.

## 4 Conclusions

We report a thermo-responsive CPL achieved by combining cellulose-based chiral nematic template of HPC with CQDs. The HPC/CQD composite material reaches high CD intensity, high CPL intensity, and high *g*_lum_ value. Most importantly, the chiral template is temperature sensitive, allowing the modulation of PBG position within the PL spectrum range of CQDs by temperature manipulation. The CPL wavelength red-shifts by 258 nm as the temperature increases from 22 °C to 44 °C, while the dissymmetry factor of CPL decreases slightly from 1.02. The decreasing chirality of CPL is due to the stronger thermal motion of HPC molecules in the longer helical pitch of cholesteric structure, which is evidenced by the control experiments with different HPC concentrations at constant temperature. On the other hand, the dissymmetry factor *g*_lum_ was found to decrease during the cooling process, to the value about 0.55, probably due to the undulation instability caused by helical pitch shrinkage.

## Supplementary Material

Supplementary Material Details
