# Supplementary material for: Thermo-responsive circularly polarized luminescence from carbon quantum dots in a cellulose-based chiral nematic template
Source: Nanophotonics. 2024 Jun 3;13(19):3679–88. doi: 10.1515/nanoph-2024-0091 (PMC11465985; doi:10.1515/nanoph-2024-0091)
Supplement: Supplementary file 1 — Supplementary Material Details [file j_nanoph-2024-0091_suppl_001.docx]

**Supplementary Materials**

Haidong Shi, ^1^ Jiaxin Zhu,^2^ Yaxuan Deng,^1^ Yanling Yang,^1^ Changxing Wang,^1^ Yihan Liu,^1^ Wanlong Zhang,^3^* Dan Luo,^2^* Da Chen,^1^* Yue Shi^1^*

**Thermo-responsive circularly polarized luminescence from carbon quantum dots in a cellulose-based chiral nematic template**

^1^ School of Physical Science and Technology, Ningbo University, No. 818 Fenghua Road, Jiangbei District, Ningbo, Zhejiang 315211, China

^2^ Department of Electrical and Electronic Engineering, Southern University of Science and Technology, No. 1088 Xueyuan Road, Nanshan District, Shenzhen, Guangdong 518055, China.

^3^ Nanophotonics Research Centre, Institute of Microscale Optoelectronics, Shenzhen University, Shenzhen 518060, China

*Author to whom correspondence should be addressed.


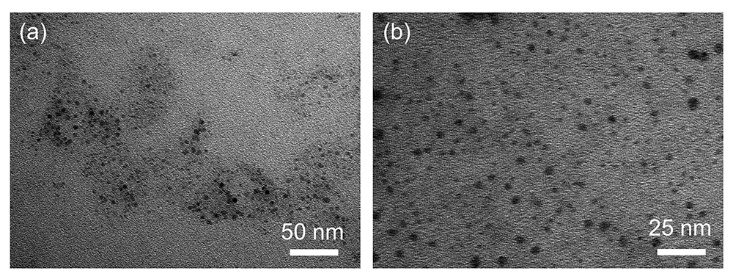


**Figure S1.** TEM images of HPC/CQD composite material with different magnifications.


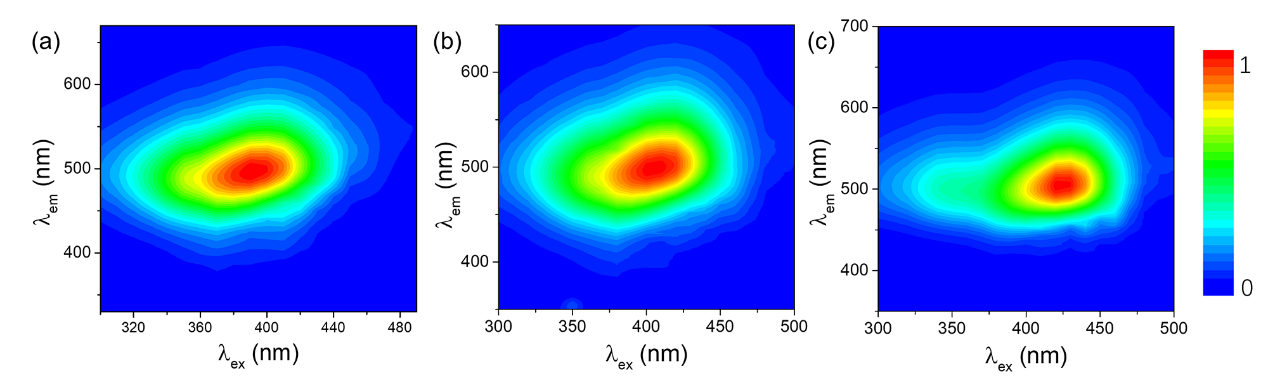


**Figure S2.** 2D excitation-emission maps of HPC/CQD materials with different CQDs contents of (a) 0.4 μg (b) 0.8 μg and (c)1.2 μg.


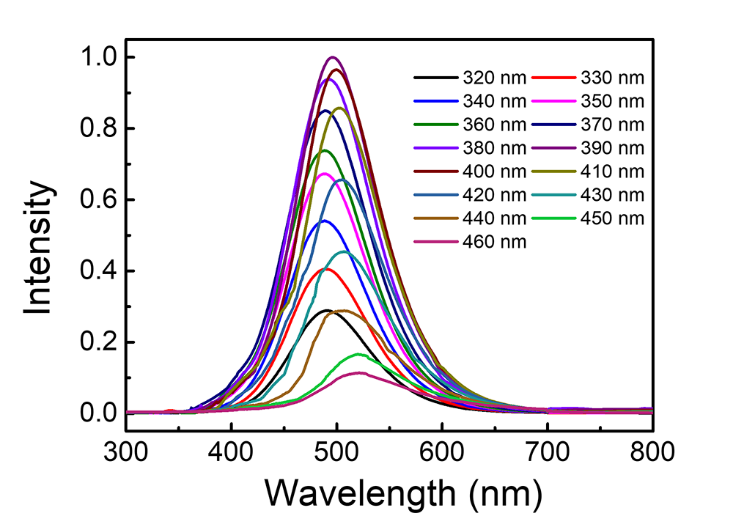


**Figure S3.** The fluorescent spectra of HPC/CQD composites containing 0.4 μg of CQDs at different excitation wavelengths.


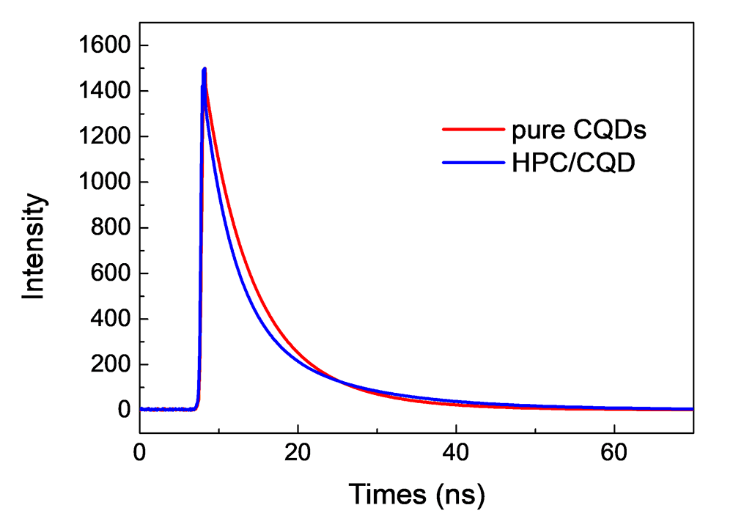


**Figure S4.** The fluorescence decay profile for the pure CQDs and HPC/CQD composite material.


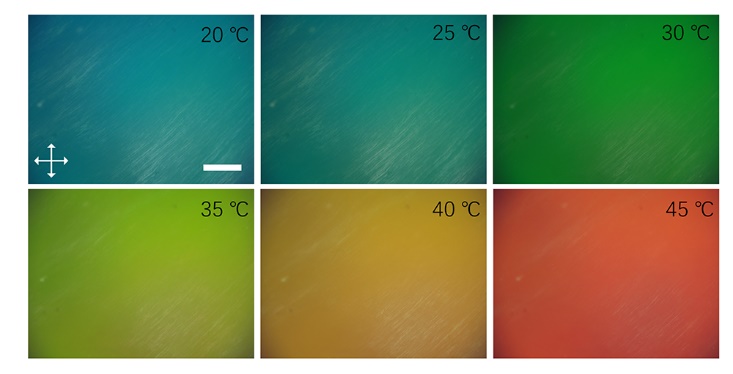


**Figure S5.** Reflected POM images of the HPC/CQD composite material at different temperatures. After rising to a specific temperature, the composite material stays for 20 min for self-assembling. The scale bar is 250 µm.


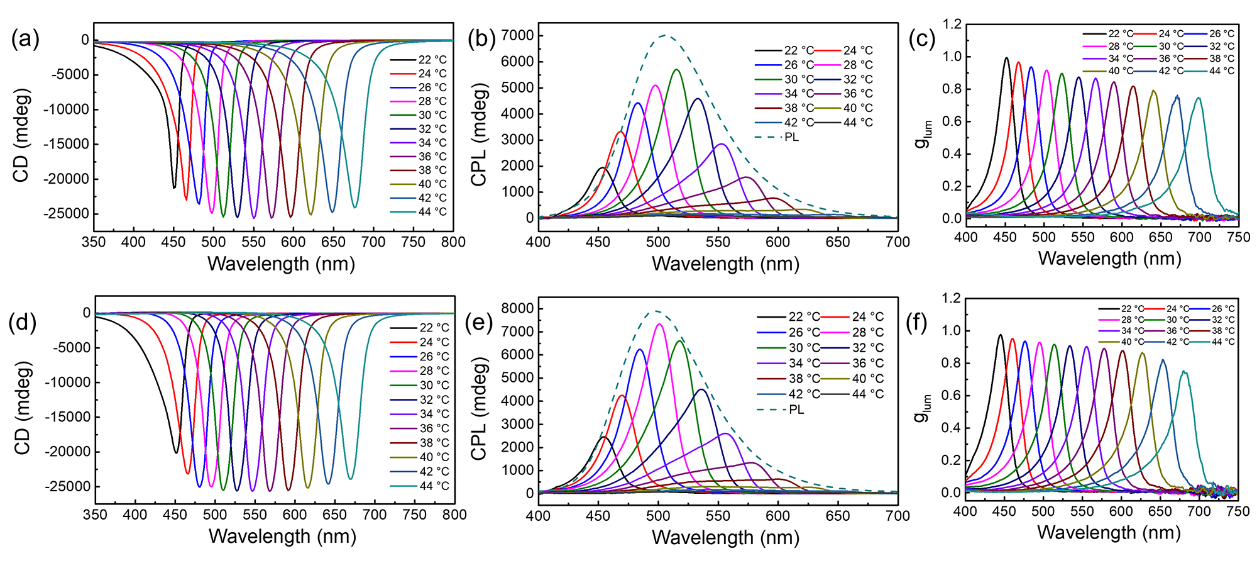


**Figure S6.** (a) CD, (b) CPL and (c) g_lum_ spectra of the HPC/CQD composite material containing 0.8 μg CQDs. (d) CD, (e) CPL and (f) g_lum_ spectra of the HPC/CQD composite material containing 1.2 μg CQDs. The excitation wavelength is 360 nm.


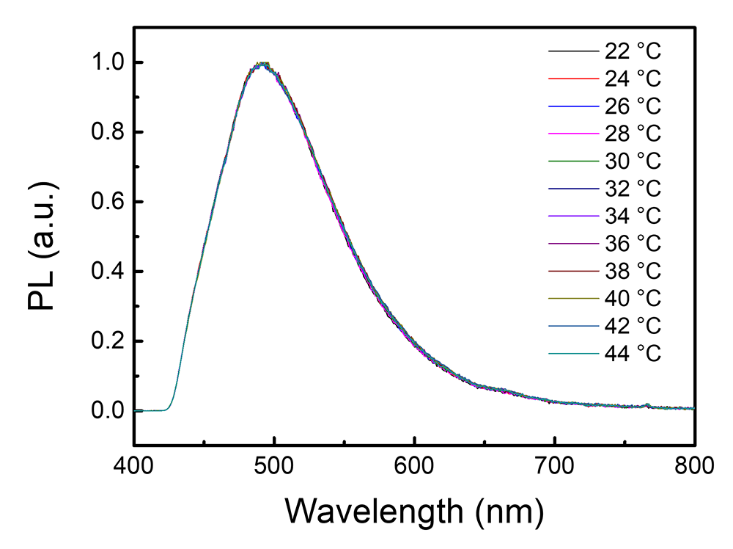


**Figure S7.** The PL spectra of CQD aqueous solution at different temperatures. The excitation wavelength is 395 nm.


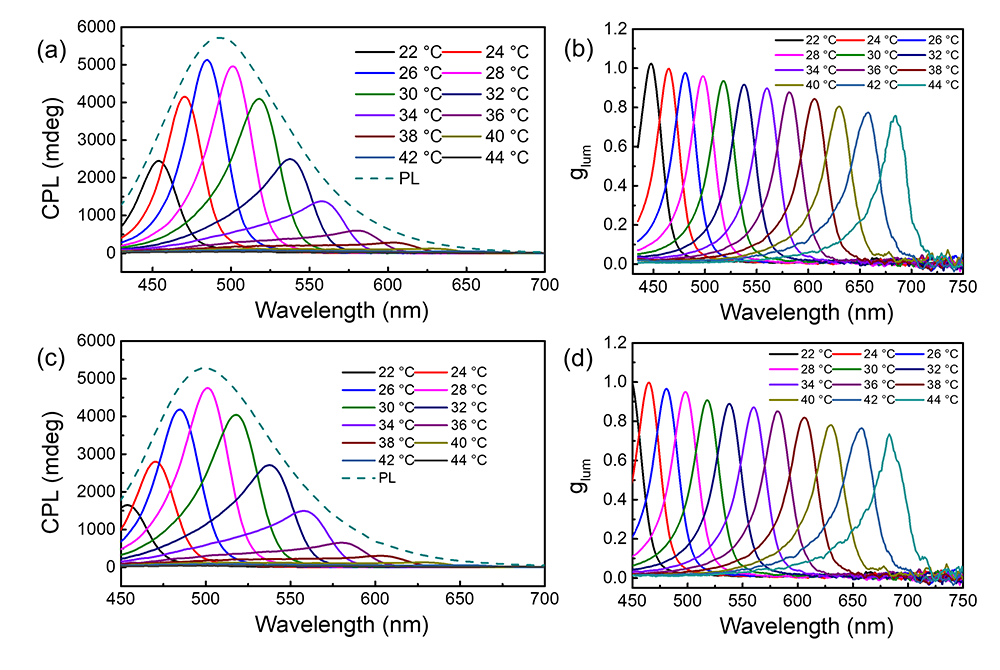


**Figure S8.** (a) CPL and (b) g_lum_ spectra with 395 nm excitation wavelength, and (c) CPL and (d) g_lum_ spectra with 410 nm excitation wavelength for the HPC/CQD composite material containing 0.4 μg of CQDs.

**Table S1:** Comparison of stimuli-responsive CPL based on chiral templates.

| Sample state | Chiral materials | Fluorescent materials | Compatibility with nanoparticles | g_lum_ and tunability | Ref. |
| --- | --- | --- | --- | --- | --- |
| low-molecular-mass LC | SLC1717+ CB-15 | AIE luminogen: TPE-PPE | No | Electrically switchable: g_lum_ = 0.4 ~ 0 | [S1] |
|  | HTW109100-000+R5011 | Upconversion nanorods | No | Electrically switchable: g_lum_ = 1.92 ~ 0  Thermally switchable: g_lum_ = 1.92 (25 ℃) vs. g_lum_ = 1.36 (109 ℃) | [S2] |
|  | SLC1717+(R)/(S)-5011 | Perovskite-polymer film | No | Thermally switchable: g_lum_ = 1.9 ~ 0 (25 ℃ ~ 85 ℃) | [S3] |
|  | PCH302/304,  PCH3E02/3E04  + (R)-/(S)-dopant | Conjugated polymer film: di-PA | No | Thermally switchable: g_lum_ = -1.30 (60 ℃) vs. g_lum_ = 1.55 (127 ℃)  g_lum_ = 1.38 (60 ℃) vs. g_lum_ = -1.59 (127 ℃)  g_lum_ peak position shifts: 460 nm ~ 625 nm (60 ℃ ~ 127 ℃) | [S4] |
|  | PCH302/304+ (R)/(S)-D1 | Chiral LC polymer film:  di-LCPA | No | Thermally switchable:  g_lum_ [λ] = -1.79 [497 nm] (25 ℃) vs. g_lum_ [λ] = 0.125 [464 nm] (40 ℃)  g_lum_ [λ] = 1.77 [510nm] (25 ℃) vs. g_lum_ [λ] = -0.146 [460 nm] (40 ℃) | [S5] |
| Supramolecular Gel | L4+SDS | Sulforhodamin B (SRB) dye | Not investigated | SRB dye molecules degrading and refueling:  g_lum_ = -0.9×10^−3^ ~ 2.3× 10^−3^ | [S6] |
| Macromolecular solid film | CNC+glycerol | Upconverting nanoparticles | Yes | Relative humidity: g_lum_ = -0.156 ~ -0.033 (RH33% ~ RH85%)  g_lum_ peak position shifts: 505 nm ~ 581 nm | [S7] |
|  | CNC | Achiral fluorescent polymer Dansyl groups | Not investigated | Acid/base switchable: g_lum_ = -0.24 ~ 0  Relative humidity switchable: g_lum_ = -0.65 ~ -0.37 (RH50% ~ RH75%)  g_lum_ peak position shifts: 450 nm ~ 700 nm | [S8] |
| Macromolecular LC | HPC | CQDs | Yes | Thermally switchable: g_lum_ = 1.02 ~ 0.8 (22 ℃ ~ 44 ℃)  g_lum_ peak position shifts: 448 nm-670 nm | Our work |

**References**

[S1] D. Zhao, H. He, X. Gu, et al., "Circularly Polarized Luminescence and a Reflective Photoluminescent Chiral Nematic Liquid Crystal Display Based on an Aggregation-Induced Emission Luminogen," *Adv. Opt. Mater.*, vol. 4, no. 4, pp. 534-539, 2016.

[S2] H. He, J. Wang, K. Li, et al., "Cholesteric-Superhelix-Enabled Reconfigurable Circularly Polarized Luminescence from Uniaxially Aligned Upconversion Nanorod Films," *Laser Photonics Rev.*, vol. 16, no. 8, pp. 2200115, 2022.

[S3] S. Liu, X. Liu, Y. Wu, et al., "Circularly polarized perovskite luminescence with dissymmetry factor up to 1.9 by soft helix bilayer device," *Matter*, vol. 5, no. 7, pp. 2319-2333, 2022.

[S4] J. Yan, F. Ota, B. A. San Jose, and K. Akagi, "Chiroptical resolution and thermal switching of chirality in conjugated polymer luminescence via selective reflection using a double-layered cell of chiral nematic liquid crystal," *Adv. Funct. Mater.,* vol. 27, no. 2, pp. 1604529, 2017.

[S5] B. A. San Jose, J. Yan, and K. Akagi, "Dynamic Switching of the Circularly Polarized Luminescence of Disubstituted Polyacetylene by Selective Transmission through a Thermotropic Chiral Nematic Liquid Crystal," *Angew. Chem. Int. Ed. Engl.,* vol. 53, no. 40, pp. 10641-10644, 2014.

[S6] H. Xu, C. S. Ma, C. Y. Yu, F. Tong, and D. H. Qu, "Reversible Inversion of Circularly Polarized Luminescence in a Coassembly Supramolecular Structure with Achiral Sulforhodamine B Dyes," *ACS Appl. Mater*., vol. 15, no. 21, pp. 25201-25211, 2023.

[S7] W. Li, M. Xu, C. Ma, et al*.*, "Tunable upconverted circularly polarized luminescence in cellulose nanocrystal based chiral photonic films," *ACS Appl. Mater. Interfaces,* vol. 11, no. 26, pp. 23512-23519, 2019.

[S8] H. Yu, B. Zhao, J. Guo, K. Pan, and J. Deng, "Stimuli-responsive circularly polarized luminescent films with tunable emission," *J. Mater. Chem. C,* vol. 8, no. 4, pp. 1459-1465, 2020.
